# Aged brain and neuroimmune responses to COVID-19: post-acute sequelae and modulatory effects of behavioral and nutritional interventions

**DOI:** 10.1186/s12979-023-00341-z

**Published:** 2023-04-12

**Authors:** Ludmila Müller, Svetlana Di Benedetto

**Affiliations:** Center for Lifespan Psychology, Max Planck Institute for Human Development, Lentzeallee 94, 14195 Berlin, Germany

**Keywords:** Aged brain, Immunosenescence, Inflammaging, Neuroinflammation, COVID-19, Long COVID, Neuroinvasion, Behavioral and nutritional intervention, Meditation, Music therapy

## Abstract

Advanced age is one of the significant risk determinants for coronavirus disease 2019 (COVID-19)-related mortality and for long COVID complications. The contributing factors may include the age-related dynamical remodeling of the immune system, known as immunosenescence and chronic low-grade systemic inflammation. Both of these factors may induce an inflammatory milieu in the aged brain and drive the changes in the microenvironment of neurons and microglia, which are characterized by a general condition of chronic inflammation, so-called neuroinflammation. Emerging evidence reveals that the immune privilege in the aging brain may be compromised. Resident brain cells, such as astrocytes, neurons, oligodendrocytes and microglia, but also infiltrating immune cells, such as monocytes, T cells and macrophages participate in the complex intercellular networks and multiple reciprocal interactions. Especially changes in microglia playing a regulatory role in inflammation, contribute to disturbing of the brain homeostasis and to impairments of the neuroimmune responses. Neuroinflammation may trigger structural damage, diminish regeneration, induce neuronal cell death, modulate synaptic remodeling and in this manner negatively interfere with the brain functions.

In this review article, we give insights into neuroimmune interactions in the aged brain and highlight the impact of COVID-19 on the functional systems already modulated by immunosenescence and neuroinflammation. We discuss the potential ways of these interactions with severe acute respiratory syndrome coronavirus 2 (SARS-CoV-2) and review proposed neuroimmune mechanisms and biological factors that may contribute to the development of persisting long COVID conditions. We summarize the potential mechanisms responsible for long COVID, including inflammation, autoimmunity, direct virus-mediated cytotoxicity, hypercoagulation, mitochondrial failure, dysbiosis, and the reactivation of other persisting viruses, such as the Cytomegalovirus (CMV). Finally, we discuss the effects of various interventional options that can decrease the propagation of biological, physiological, and psychosocial stressors that are responsible for neuroimmune activation and which may inhibit the triggering of unbalanced inflammatory responses. We highlight the modulatory effects of bioactive nutritional compounds along with the multimodal benefits of behavioral interventions and moderate exercise, which can be applied as postinfectious interventions in order to improve brain health.

## Introduction

Over the course of the Corona pandemic, in addition to a SARS-CoV-2 infection, such conditions as social isolation, feelings of a lack of control, uncertainty, loneliness, and fear of infection contributed—particularly among the elderly population—to mental and psychological problems including acute and chronic stress, anxiety, and depression. Much work has demonstrated that the immune system plays a decisive role not only in response to infection but also in the regulation of the extremely complex neuronal and social processes. It appears that the brain and the immune system act in concert, responding to external challenges by means of mutual and extremely entangled neuroimmune interactions to maintain the internal homeostasis of the body. Both of these systems stand in a constant cross-talk to each other in order to facilitate an optimal response of the organism to changing internal and external environmental stimuli [1, 2].

During the Corona pandemic, particularly the elderly, who are most predisposed to chronic neurogenerative diseases, have also been confronted with physical inactivity, sociopsychological stress and worsened nutritional conditions in addition to a COVID-19 infection [3–6]. These pandemic-related disturbances may potentiate the already existing age-associated physiological changes in neuroimmune processes and negatively affect the course of COVID-19, leading later to the development of post- and long COVID symptomatology in this group of patients.

Recently, there has been increasing evidence that the immune system, particularly through inflammatory cytokines, can significantly influence stress circuits of the central nervous system (CNS), together with hormonal, and neurochemical responses [2]. Brain regions affected by cytokines include the amygdala, which is important in threat appraisal, as well as the hippocampus, cingulate cortex, and prefrontal cortex [1, 7, 8]—all of which are involved in the shaping of social behavior and the formation of highly plastic learning processes to ensure survival. Thus, pro-inflammatory cytokines appear to act as soluble mediators of neural plasticity and influence the circuits that could be essential for neurological processes [2]. If unchecked—as in the case of COVID-19 infection—these cytokines can induce enormous biological dysregulations, including the brain tissue injury and have negative consequences across multiple organ systems [9].

Age-related neuroinflammation accompanied by an increased release of pro-inflammatory cytokines and cortisol may additionally accelerate the progression of various neurodegenerative and neuropsychiatric complications caused by virus-related pathological activity and hyperinflammation, leading to further neuroimmune inflammatory responses by oxidative damage to cells, proteins, lipids, and DNA in the brain. As a result of these cumulative effects, the decline in brain function and lasting brain tissue damage occurs leading to the loss of life autonomy in the elderly [5]. Additionally, neuropsychiatric complications, such as anxiety, depression, traumatic stress disorder, insomnia, etc., related to COVID-19 were reported to be extremely common and may negatively impact the quality of life [10].

In this review article, we consider the neuroimmune interactions and the impact of COVID-19 on an aging organism in which the virus is encountering functional systems already modulated by immunosenescence and neuroinflammation. We highlight the potential ways of these neuroimmune interactions with SARS-CoV-2 and give insights into proposed neuroimmune mechanisms and biological factors that may contribute to the development of persisting long COVID conditions. Although the long-term relationships between COVID-19 and neurophysiological consequences will take years to surface, the detailed understanding of the role of immunosenescence and neuroinflammation as well as the differentiation of underlying mechanisms in neuroimmune responses to SARS-CoV-2 infection would be an important step towards developing therapeutics and potential ways of interventions.

## The impact of immunosenescence and inflammaging on the immune response to SARS-CoV-2

Advanced age is one of the significant risk factors for COVID-19-related mortality and for long COVID complications, which are currently a subject of increasing attention playing a crucial role in the life of many people after a bout of infection. What are the reasons allowing the virus to escape an immune response and to produce such dramatical pathological changes in all physiological systems including the brain? The contributing factors can be on the one hand, the age-related dynamical remodeling of our immune system, known as immunosenescence, that is characterized with the reduced or impaired function of adaptive and innate immunity. On the other hand, chronic subclinical systemic inflammation, known as inflammaging [11], also plays a decisive role in the development of many age-related disorders and may contribute to the pathology of COVID-19.

The etiology of inflammaging is not fully understood, but both cell-endogenous and exogenous factors and physiological stressors are possible contributors to a chronic age-related inflammation [12]. The accumulation of senescent cells producing inflammatory cytokines, reactive oxygen species (ROS), metalloproteinases and fibronectin may contribute to inflammaging. Pro-inflammatory factors, such as interleukin (IL)-6, IL-1β, the C-reactive protein, and the tumor necrosis factor (TNF), may also be released from the visceral adipose tissue [13] of aged individuals. The activation of the immune inflammatory cells may occur due to the microbial dysbiosis [14], but also due to the disturbed proteostasis, leading to an accumulation of misfolded proteins and cellular debris [15]. These fragments of cellular garbage may serve as ligands for certain pattern recognition receptors initiating pro-inflammatory signaling [16]. Thus, in combination with other contributing factors, both immunosenescence and inflammaging may lead to a poorer antiviral immune response and disturbed viral clearance and also to an increased risk of immune dysregulation [9].

**Fig. 1:**
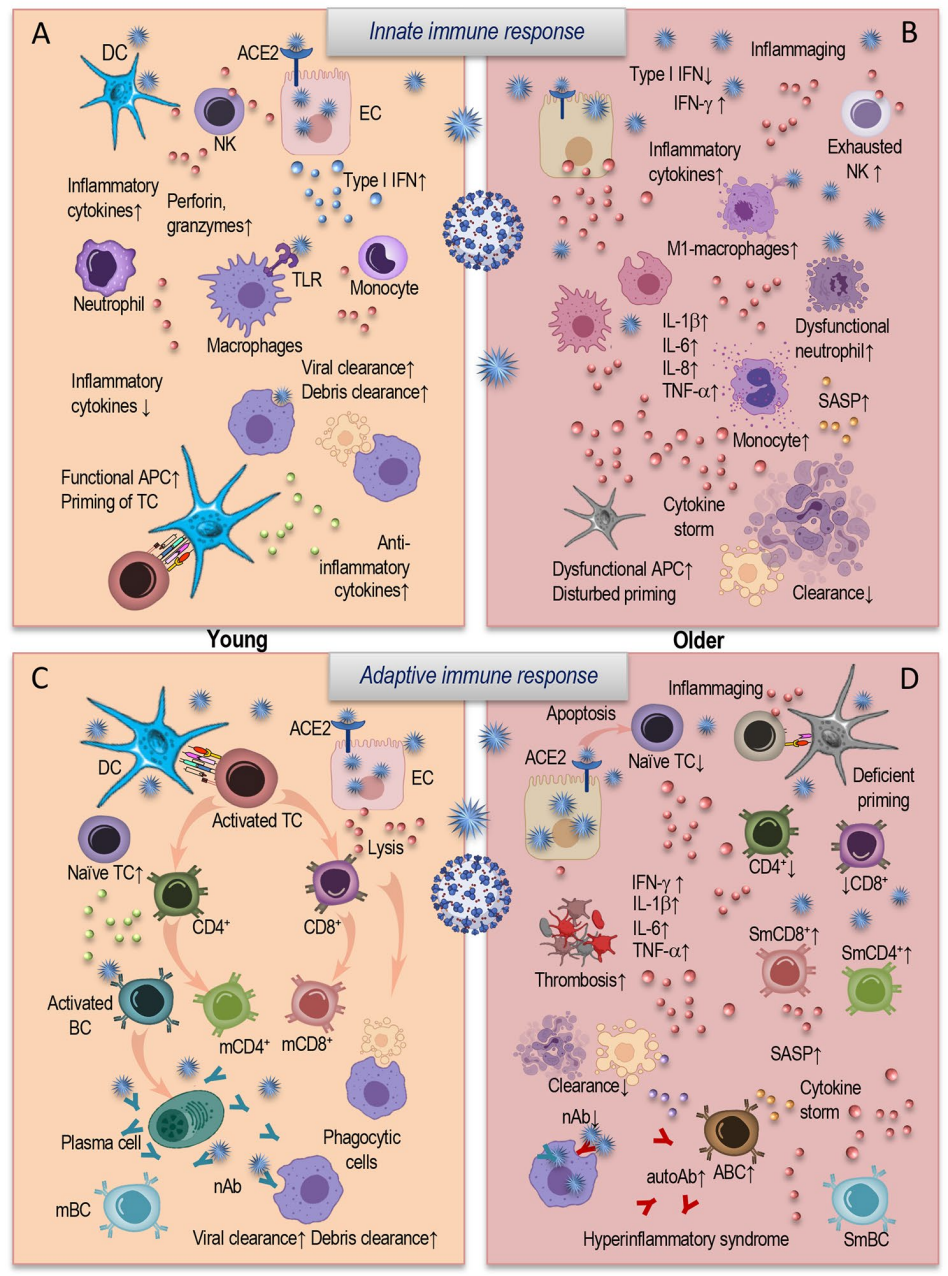
The impact of age-related changes in the innate and adaptive immune system on COVID-19. The SARS-CoV-2 infects the cell by binding to the ACE2 receptor. After invasion, the virus can be detected by the cells of innate immunity (**A, B**), such as monocytes, macrophages, and DC. In young (**A**) it leads to an induction of a local inflammatory and robust interferon-I response, inhibiting the viral replication. Immune cells are recruited to the site of infection: NK cells kill infected cells, neutrophils clear the cell debris, and the functional APCs prime cells of the adaptive immunity (**C**). After activation, the CD4^+^ T cells release cytokines and activate B cells and plasma cells to produce the nAb. Cytotoxic CD8^+^ T cells can directly kill infected cells, preventing the viral spread. Neutrophils migrate to the sites of infection to clear the cell debris. The immune response of young individuals (**A, C**) efficiently resolves the infection and is capable of establishing an immune memory. The innate cells of elderly individuals (**B**) are functionally impaired and enable to induce robust antiviral type I IFN response for controlling virus replication. Inflammatory and SASP molecules attract further dysfunctional inflammatory neutrophils, monocytes, and inflammatory M1-macrophages to the sites of infection, establishing an inflammatory feedback loop and contributing to the so-called “cytokine storm”. These detrimental conditions inhibit effective T-cell priming and disturb efficient debris clearance. Aged cells of the adaptive immune system (**D**) also have multiple deficits, which prevent an effective antiviral immunity. Decreased T-cell numbers with reduced receptor repertoire, an accumulation of senescent T cells with impaired proliferative capacity, and elevated levels of inflammatory cytokines lead to the disturbed immune response to the SARS-CoV-2. Senescent B and T cells produce inflammatory cytokines, inhibiting the generation of mature B cells. Reduction in nAb and elevation of non-neutralizing antibodies and autoAb may augment the SARS-CoV-2 infection by ADE, leading to organ damage. Autoimmunity, SASP, and inflammaging promote a pro-thrombotic environment and contribute to the hyperinflammatory syndrome observed in severe COVID-19. Abbreviations: DC: Dendritic cell; NK: natural killer cell; EC: endothelial cell; ACE2: angiotensin-converting enzyme 2; IFN: interferon; TLR: toll-like receptor; IL: interleukin; TCR: T-cell receptor; SASP: senescence-associated secretory phenotype; APC: antigen-presenting cell; TNF: tumor necrosis factor. SmCD8+: senescent memory CD8^+^ T cell; SmCD4^+^: senescent memory CD4^+^ T cell; SmBC: senescent memory B cell; ABC: age-associated B cells; ADE: antibody-dependent enhancement. Modified from [9].

The disparities in the immune function between young and older individuals have been dramatically reflected in the differential immune responses to SARS-CoV-2 (Fig. 1). The exceptional ability of a young immune system is characterized by initialization of an immediate innate immune response (Fig. 1, A) after the recognition of SARS-CoV-2 RNA and viral protein components through pattern recognition receptors—such as toll-like receptor (TLR). The resulting local inflammation and type I IFN antiviral responses allow for the inhibition of viral replication [9, 17]. This process is accompanied by the recruitment and activation of immune cells, resulting mostly in the induction of an effective specific immunity, capable of eliminating the virus and contributing to the accomplishment of a stable, successful clinical recovery (Fig. 1, A and C).

Immunosenescence affects the proportions and functional capabilities of immune cells and predisposes the elderly to an inappropriate immune response to SARS-CoV-2 infection (Fig. 1, B and D). The immune response of an aged host fails to launch a robust type I IFN antiviral response to control the SARS-CoV-2 viral replication (Fig. 1, B). The neutrophil function is also impaired, so that these immune cells are unable to efficiently eliminate the pathogen immediately after entry. In addition, neutrophils are capable of escaping apoptosis, but can produce elevated amounts of inflammatory molecules [87]. In fact, the increased cell counts of neutrophils and monocytes in the blood of COVID-19 patients are indicative of a negative prognosis and are often associated with a severe course of the disease [12, 18, 19].

The accumulation and persistence of senescent immune cells that produce excessive amounts of inflammatory cytokines, have an abnormal senescence associated secretory phenotype (SASP), and are resistant to apoptosis—is one of the main features of immunosenescence and inflammaging. The cells with SASP are able not only to produce pro-inflammatory cytokines, chemokines, fibronectins and matrix metalloproteinases, but also to negatively affect the other cells by further enriching the environment with pro-inflammatory mediators and reactive oxygen species. Moreover, the increase in the number of senescent immune cells is accompanied by the decreased ability of the immune system to remove these senescent cells, leading to a further enrichment of the microenvironment with inflammatory molecules and further cell damage [9, 20, 21]. Thus, SASP mediators along with a disproportional presence of inflammatory cells may trigger the exaggerated hyperinflammatory conditions in the elderly and be one of the mechanisms for an excessive inflammation during COVID-19 infection [12, 22].

The age-related changes in the important function of innate immune cells concerning their antigen presentation negatively influence the priming and activation of the T cells. The monocytes and dendritic cells from elderly people demonstrate a reduced expression of CD40, CD86 and MHC class II molecules [21, 23]. It has been shown that innate cells from patients with acute COVID-19 also exhibited reduced antigen-presenting capacities and a low expression of human leukocyte antigen (HLA)-DR, CD80, and CD86 when stimulated in vitro [24]. Thus, a SARS-CoV-2 infection may further negatively influence the cells of the aged innate immune system towards dysregulation in their main function of priming the adaptive immune system [9, 12].

The effective antiviral immune response may also be disturbed due to the multiple age-related impairments in cells of the adaptive immune system (Fig. 1, D). The age-related loss of T- and B-cell diversity may limit the efficient response to such a novel pathogen as SARS-CoV-2 [25]. The accumulation of CD28^−^ T cells in the peripheral circulation expressing multiple senescence markers inhibits the necessary secondary T-cell activation signaling and may prevent the antiviral T-cell response [21, 26–28]. It was also supposed that SARS-CoV-2 spike proteins may induce the apoptosis of T cells by direct interaction with T cells through CD26 surface molecules [29], thus decreasing T-cell immune response.

Age-related impairments in the functional capacity of CD4^+^ T cells to trigger B cells through the cytokine production for differentiation into immunoglobin-producing plasma cells [30] may also influence the humoral response to the virus. It was shown that proportions of virus-specific T cells correlate with serum titers of IgG and IgA [31]. Age-associated changes in the immunoglobulin class-switch recombination and somatic hypermutation may also have a negative impact on the production and secretion of high-affinity antibodies that play an important role in the establishment of protective and long-lasting immunity [9, 32, 33] to the virus.

Due to age-related chronic low-grade inflammation, elevated senescent cell load, SASP and inflammasome activation, increased DNA damage, and reduced autophagy—an induction of the overwhelmed pathological inflammatory responses may occur in aged patients [9, 34]. Such a highly inflammatory environment and predisposition to autoimmunity may also start the prothrombotic pathways supporting thrombosis and contributing to further inflammation in a feed-forward loop. Thus, immunosenescence and inflammaging in combination with a SARS-CoV-2 infection may additionally induce a prothrombotic environment, stimulate a hyperinflammatory immune response, and negatively influence the course of COVID-19 in aged patients [9]. The hyperinflammation, as a result of an abnormal immune and inflammatory reactions, may induce a pathological damage to many physiological systems and be one of the possible contributors to the development of a wide range of chronical neurological complications in COVID-19 survivors with long-term consequences for them.

## Aged brain and neuroinflammation

The vulnerability of the aged brain could not only originate from the impaired immune defenses but also from any of the altered homeostatic mechanisms that contribute to the aging phenotype. One of such critical changes in the aged brain involves the age-related alterations in the microenvironment of neurons and microglia, which are characterized by a general condition of low-grade inflammation—so-called neuroinflammation. Especially changes in microglia, which represent the brain resident macrophage cells playing a decisive regulatory role in inflammation, contribute to disturbing of the brain homeostasis [2, 35].

**Fig. 2:**
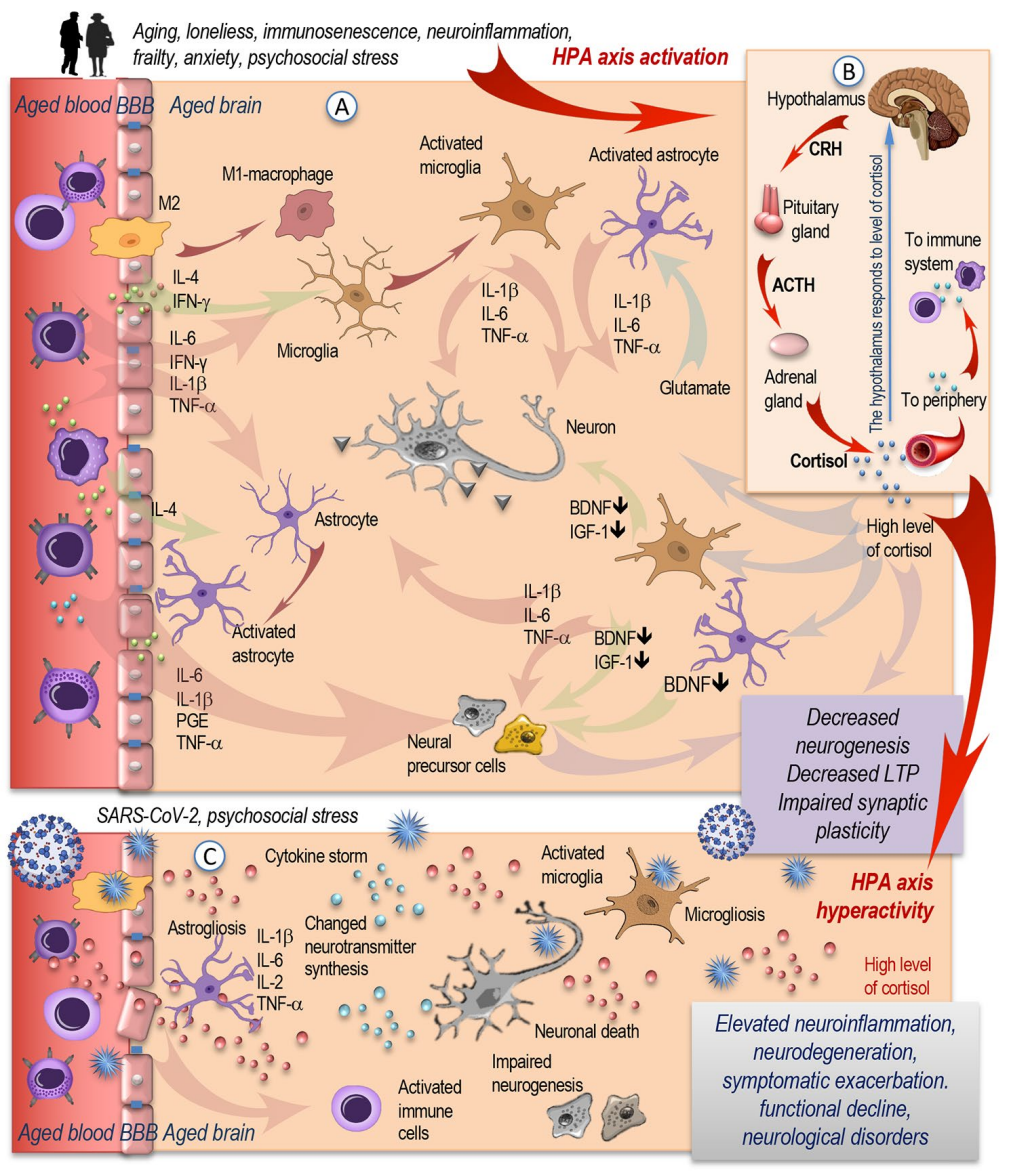
The aged brain, neuroinflammation, and the impact of SARS-CoV-2. (**A**) Aging, peripheral immunosenescence, and inflammaging induce age-related changes in the blood. Chronic exposure to pro-inflammatory factors may disrupt the endothelial barrier and allow the unhindered transfer of immune cells and pro-inflammatory cytokines into the brain parenchyma, activating microglia and driving low-grade brain inflammation. Activated microglia and astrocytes produce further inflammatory mediators. Protective M-2 macrophages turn into pro-inflammatory M1-phenotype and contribute to further neuroinflammation. An inflammatory environment disrupts the delicate balance needed for LTP-induction, impairs synaptic plasticity, and downregulates the production of BDNF and IGF-1. This leads to negative consequences for neural precursor cells decrease and for the normal neuronal functioning. (**B**) Aging, stress, and inflammaging activate the HPA axis to release CRH from the paraventricular nucleus and trigger the anterior pituitary gland to secrete ACTH. This stimulates the release of glucocorticoids from the adrenal gland into the circulation. High concentrations of cortisol can, in turn, impair hippocampal neurogenesis. (**C**) Infection with SARS-CoV-2 may be an additional immune stressor, contributing to an elevated neuroinflammatory markers and resulting in more intense and unremitting immune reactions. The SARS-CoV-2-induced astro- and microgliosis contributes to BBB-disintegration and elevated levels of pro-inflammatory cytokines and is associated with neuronal loss. These combined effects may lead to symptomatic exacerbation, neurodegeneration, and a perpetuation of functional decline Abbreviations: SARS-CoV-2: Severe acute respiratory syndrome coronavirus type 2; HPA: Hypothalamic-pituitary-adrenal axis; CRH: corticotropin releasing hormone; ACTH: adrenocorticotropin; IL: interleukin; IFN: interferon; TNF: tumor necrosis factor; LTP: long term memory potentiation; BDNF: brain-derived neurotrophic factor; IGF: insulin-like growth factor. Modified from [2].

The underlying age-related conditions in the blood, including low-grade inflammation and immunosenescence, may on a systemic level contribute to the neuroinflammation in the aged brain. Emerging evidence reveals that the immune privilege in the aging brain may be compromised [36, 37]. An excess of soluble inflammatory mediators including cytokines (e.g., TNF-α, IL-1β, IL-6, IFN-γ), pathogen-associated molecular pattern molecules (e.g., LPS, viral nucleic acids), complement components, sphingosine, prostaglandins, and kinins may negatively influence the blood-brain barrier (BBB) [38]. The endothelial barriers can be disrupted under the influence of persistent exposure to inflammatory mediators, allowing for the entrance of immune cells and the unhindered transfer of inflammatory cytokines into the brain parenchyma (Fig. 2, A). This may induce an inflammatory milieu and drive the low-grade inflammation in the brain tissue by modulating and activating microglia to produce further inflammatory cytokines.

Resident cells, such as astrocytes, neurons, oligodendrocytes and microglia, but also infiltrating immune cells, such as monocytes, T cells and macrophages participate in the complex intercellular networks and multiple reciprocal interactions between activated cell surface receptors and secreted inflammatory mediators and cytokines, thus promoting the process of neuroinflammation [2, 36, 37, 39–41]. Neuroinflammation may trigger structural damage, diminish regeneration, induce neuronal cell death, modulate synaptic remodeling, and, in this manner, negatively interfere with the brain functions.

The pro-inflammatory environment drives morphological and functional alterations of astrocytes and microglia, diminishing their neuroprotective functions and inhibiting the neurogenesis. It is supposed that microglia may experience age-associated changes, characteristics of which are similar to the peripheral immune cells [9, 42]. The aged brain cells, on the other hand, may also modulate the immune system and contribute to the recruitment of immune cells from the periphery, thereby promoting further immunosenescence and neuroinflammation [36]. It was reported that aged microglia were able to recruit circulating CD8^+^ T cells that entered the brain with the help of the adhesion molecules anchored on the surface of the brain endothelial cells [43]. The accumulation of CD8^+^ T cells in the aged brain has been shown to increase the production of IFN-γ and to have detrimental effects on the neural stem cell function [40]. An increased amount of these T cells in the brain of aged mice has been associated with axon degeneration and with age-related cognitive and motor decline [39].

It is known that psychosocial stress, loneliness, anxiety, and aging itself can jointly distress the neuroendocrine system, by stimulating the hypothalamic-pituitary-adrenal (HPA) axis to produce the corticotropin-releasing hormone (CRH) from the hypothalamus and thus trigger the anterior pituitary gland to release adrenocorticotropin (ACTH) (Fig. 2, B). This results in the production and release of glucocorticoids (such as cortisol) from the adrenal gland into circulation [44]. High levels of cortisol can directly diminish hippocampal neurogenesis or indirectly by modulating the release of cytokines and neurotrophins and altering the expression of their receptors on the surface of immune and brain cells. These changes may lead to impairments in synaptic plasticity, to prolonged neuroinflammation, and age-related neurobehavioral disturbances [2].

It is supposed that, at least in rodents, levels of inflammatory cytokines increase as a function of age [44–46] and that aging microglia develop a primed profile, which Norden and colleagues defined as “(i) an increased baseline expression of inflammatory markers and mediators; (ii) a decreased threshold `to be activated and to switch’ to a pro-inflammatory state; and (iii) an exaggerated inflammatory response following immune activation” [7]. Thus, aging and neuroinflammation can sensitize the aged brain to produce an exaggerated response following exposure to a stressor and to the presence of an immune stimulus in the periphery [2, 7, 47–49]. The SARS-CoV-2 may represent such an immune stressor and, in addition to the age-related neuroinflammation, impact the aged brain (Fig. 2, C). The inadequate systemic hyperinflammation can also disrupt brain homeostasis and have adverse effects on neuronal cell functions, leading to behavioral and cognitive impairments and triggering COVID-19 neuropathology [1, 8].

It has also been suggested that susceptibility of the brain to the cytokine storm in COVID-19 may be related to “microglial priming” as a result of age-related neuroinflammation [50]. Such microglial priming may produce an exaggerated microglial response and induce a positive feedback loop in which more cytokines and inflammatory mediators are produced. This may contribute to the elevation of neuroinflammatory markers, the aggravation of neuroinflammation, and result in a more intense and unremitting immune response. In the brain parenchyma, the unrestricted inflammatory reactions are enormously potent to initiate injury cascades, leading to brain tissue damage and functional dysregulation; affecting neurogenesis, synaptic neurotransmission and plasticity, mitochondrial functioning and brain homeostasis [51, 52]. Brain tissue injury can also occur as a result of microbleeds caused by SARS-CoV-2-induced endothelial damage. The virus can trigger astrogliosis that may additionally contribute to a BBB-disintegration due to the progressive foot detachment of astrocytes. The SARS-CoV-2-induced microgliosis promotes microglia to secrete elevated levels of cytokines. Additionally, both microgliosis and astrogliosis are associated with neuronal loss [51].

Both, inflammatory cytokines that enter the brain and locally induced neuroimmune inflammatory responses can influence the production, release, and metabolism of several important neurotransmitters, including dopamine, norepinephrine, and serotonin [2, 53]. Such alterations in the metabolism and in the levels of neurotransmitters are known to be responsible for the pathophysiology of various neuropsychiatric conditions, such as anxiety, depression, and obsessive-compulsive disorders [54, 55]. As fluctuations in cytokine levels can lead to a disturbance in the metabolism of neurotransmitters and be responsible for triggering behavioral disorders, it has been hypothesized than neuroimmune interactions can be placed as a critical link between a SARS-CoV-2 infection and mental health impairment [56]. Therefore, all these effects may jointly lead to symptomatic exacerbation and the perpetuation of functional decline, particularly in exposed elderly individuals [1, 57].

## Possible pathways and mechanisms of brain invasion

Along with neuroinflammation, leading to a break-down of the brain homeostasis, there is a growing body of evidence indicating multidimensional pathways of a brain invasion by SARS-CoV-2. Although the exact mechanism of brain invasion is not fully understood, several routes of viral entry have been proposed, including a hematogenous route by peripheral immune cells of the bloodstream as well as trans-neuronal routes of invasion [1, 58, 59].

It is widely known that SARS-CoV-2 binds to the receptor for the angiotensin-converting enzyme 2 (ACE2) [60–62] and therefore, the expression of this receptor dictates the entrance point of the virus. While ACE is most prominently expressed by epithelial and endothelial cells, it has been found to be expressed, to a lesser extent, by neurons and glial cells [61]. The viral spike protein interacts with the ACE2 receptor and induces an increase of angiotensin II, activating the nicotinamide dinucleotide phosphate oxidase 2 (NOX2) enzyme with the subsequent release of reactive oxygen species (ROS) and inflammatory mediators in the central nervous system [63]. Due to the high expression of ACE2 receptors on the endothelium of brain blood vessels [64], they may operate as docking sites for the virus and promote its hematogenous dissemination.

**Fig. 3:**
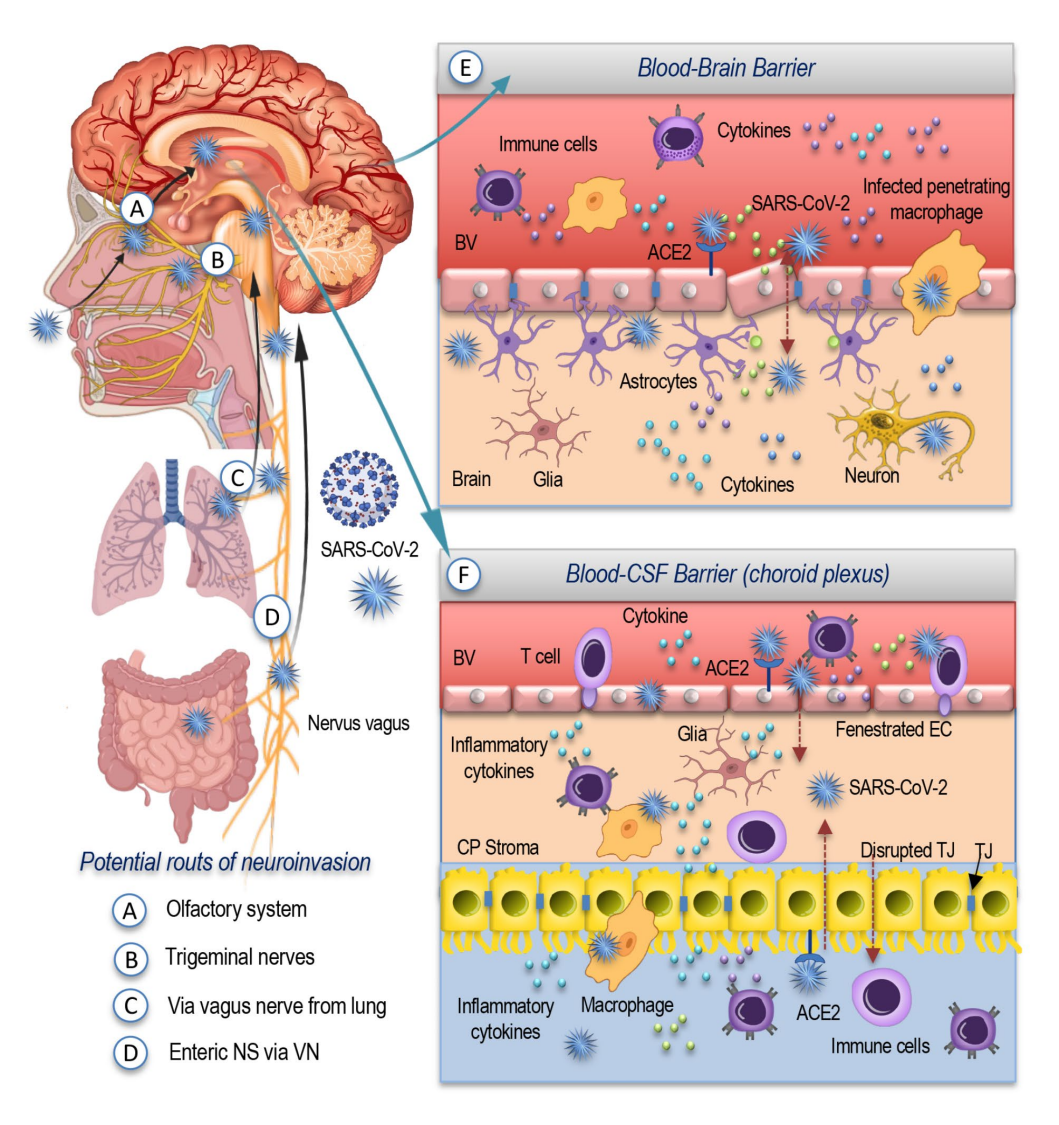
Possible routes of brain invasion. SARS-CoV-2 may enter the CNS through the olfactory system (**A**) cross the neural-mucosal interface in the olfactory mucosa, infect the olfactory neurons and then migrate up to the medulla oblongata. The virus can also take a route through the trigeminal nerve (**B**), infecting it in the nasal cavity. Other potential routes of neuroinvasion by SARS-CoV2 include pathways of retrograde synaptic transport via axons from receptors in the lung (**C**) and via the vagus nerve from enteric nervous system (**D**). The brain invasion may occur by disrupting the BBB (**E**), and by compromising the function of the choroid plexus (**F**). This can lead to the triggering of a neuroinflammatory response and promote the cerebral inflammatory state Abbreviations: SARS-CoV-2: severe acute respiratory syndrome coronavirus type 2; ACE2: angiotensin-converting enzyme 2; TJ: tight junctions; BV: blood vessel; CP: choroid plexus; EC: epithelial cell; CSF cerebrospinal fluid; NS: nervous system; VN: vagus nerve.

There are many uncertainties regarding the possible pathways of viral transfer, but high prevalence of anosmia in patients with COVID-19 allows to suppose the involvement of the olfactory system as one of the most potential routes of neuroinvasion (Fig. 3, A). The direct infection of the olfactory bulb may occur by the virus attaching to the olfactory nerve terminals, becoming internalized, and then being transported to other regions of the brain [65]. Due to the fact that the impairments in olfaction and taste were usually reported at the beginning of the COVID-19 infection [65], it was suggested that the virus may be able to enter the CNS by retrograde axonal travel through the cribriform plate [66]. This means that SARS-CoV-2 may be capable of crossing the neural-mucosal interface in the olfactory mucosa, of infecting the olfactory neurons, and then of migrating up to the medulla oblongata [58], causing neuroinflammation, demyelination, and neuronal loss [67].

Another less discussed potential route of viral invasion is the entry through the trigeminal nerve (Fig. 3, B) that might be affected together with the olfactory system. Along with prominent olfactory dysfunction, this could also lead to such common neurological symptoms, like headaches, in COVID-19 patients [68]. The possible suggested mechanism for these impairments may be the direct invasion and infection of the trigeminal nerve in the nasal cavity by SARS-CoV-2 [69–71], which can also be due to the fact that the intranasal olfactory and trigeminal systems possess wide interconnections and a close relationship [58].

Other suggested potential routes of neuroinvasion by SARS-CoV-2 include pathways of retrograde synaptic transport via axons from receptors in the lung (Fig. 3, C) and via the vagus nerve from enteric nervous system (Fig. 3, D) into the respiratory areas within the medulla of the brainstem [64, 72]. Small intestine endothelial cells are characterized by a high expression of ACE2 and are involved, along with neurons, in the enteric nervous system. In COVID-19 patients, the gastrointestinal symptoms are frequently present [73, 74]. It has been reported that SARS-CoV-2 was able to effectively replicate inside enterocytes [75] and could be isolated not only from oral but also from anal swabs [76].

The hematogenous route of neuroinvasion may be used by SARS-CoV-2 via infected lymphocytes that cross the blood-brain barrier (Fig. 3, E) smuggling the virus into the brain or by direct infection of ACE-expressing microvascular endothelial cells [77]. The ACE2 expression is lower in the brain compared to other tissues and organs, but, nevertheless, the high expression has been detected in the choroid plexus and paraventricular nuclei of the thalamus. The nuclear expression of ACE2 was evident in neuronal as well as non-neuronal cells, such as endothelial cells, astrocytes, oligodendrocytes, in the posterior cingulate cortex, and middle temporal gyrus [78, 79].

The virus may perform the neuroimmune effects either by directly entering the intracellular compartment of glia and neuronal cells, or by producing the secondary damage by inflammatory mediators of both, systemic origin or derived from inflammatory resident neuroimmune cells (Fig. 3, E and F). In fact, some recent critical evidence suggest that CNS effects might be possibly due to the transmission of inflammatory mediators from the choroid plexus [80] at the level of the blood-CSF (cerebrospinal fluid) barrier (Fig. 3, F) rather than to be caused by the virus penetrating the brain parenchyma. The authors provide the argument that they failed to detect the virus RNA or protein in the gene expression profiles from the choroid plexus and medial prefrontal cortex of individuals, who died from COVID-19. But, on the contrary, they were capable of revealing evident alterations in various inflammatory genes [80]. Another research group however, reported that viral RNA has been detected in the olfactory mucosa and in the uvula and medulla oblongata by using RT-qPCR and in-situ hybridization to detect the SARS-CoV-2 RNA as well by means of immunohistochemistry and electron microscopy [58].

Thus, the different mechanisms or a combination of them may support the neuroimmune invasion involving the viral neurotropism or direct viral entry by compromising the choroid-plexus, by disrupting the BBB, by triggering the inflammatory response, and by promoting the cerebral inflammatory state.

## Potential mechanisms that may contribute to long COVID

Residual symptoms after a SARS-CoV-2 infection, which are observed in both, the severe and non-severe disease cases, are concerning. These conditions have various names, such as “post-acute coronavirus disease (COVID) syndrome” (PACS), “post-COVID19 syndrome”, or “post-acute sequelae of SARS-CoV-2 infection” (PASC) but are more generally known as “long COVID”. The long COVID disease is defined as a cluster of symptoms lasting more than 28 days after an acute COVID-19 infection. This is an umbrella term for a spectrum of symptoms including mostly anxiety, chronic fatigue, so-called brain fog, concentration disorders, attention and memory deficits, changes in mood, and insomnia. Approximately 30% of patients develop long COVID after a SARS-CoV-2 infection and various neuroimmune mechanisms seem to be involved in the pathogenesis of this syndrome [81].

**Fig. 4:**
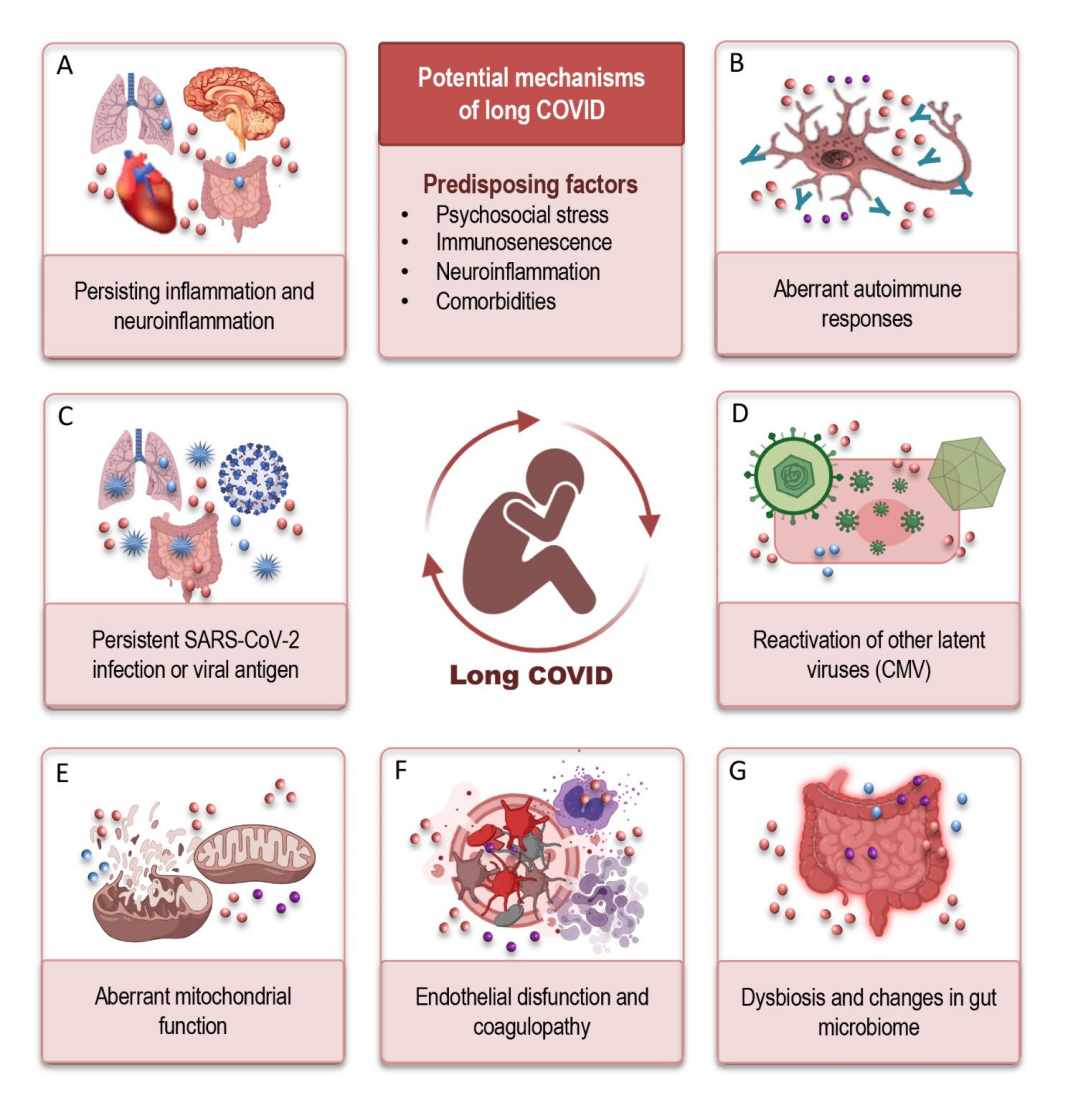
Potential mechanisms of long COVID. The interplay of multiple neuroimmune and SARS-CoV-2-specific potential mechanisms, including persisting inflammation (**A**), autoimmunity (**B**), direct virus-mediated cytotoxicity (**C**), and the reactivation of other persisting viruses, such as CMV (**D**), aberrant mitochondrial function (**E**), endothelial disfunction, and hypercoagulation (**F**), dysbiosis and changes in microbiome (**G**) may operate in various combinations following SARS-CoV-2 infection and contribute to long COVID. The inflammation (red dots) seems to be a core component in all these putative mechanisms, inducing disturbed neuroimmune responses and leading to a persistent physiological and neurological alterations, especially but not exclusively in older population Abbreviations: COVID: coronavirus disease; SARS-CoV-2: severe acute respiratory syndrome coronavirus type 2, CMV: cytomegalovirus.

Due to the relatively new encounter of the long COVID, knowledge about mechanisms and biological factors contributing to this chronic disease is incomplete, but rapidly progressing. Growing evidence revealed that a concerted action of the viral and host biological factors may be responsible for the development of the persistent long COVID symptoms [82, 83]. It was hypothesized that an interplay of multiple neuroimmune and SARS-CoV-2-specific potential mechanisms (Fig. 4, A-G) including persisting inflammation, autoimmunity, direct virus-mediated cytotoxicity, hypercoagulation, mitochondrial failure, dysbiosis, and a reactivation of other persisting viruses, may contribute to pathologic impairments in various physiological systems including the brain.

Persisting inflammation (Fig. 4, A) has been recognized as a crucial factor in the pathogenesis of long COVID. The pro-inflammatory mediators may play a central role in the pathophysiological mechanisms driving long COVID symptomology. Elevated inflammatory markers were measured for several months [84, 85] in patients with long COVID compared to fully recovered patients [85, 86] and were associated with cognitive impairments. In post-COVID patients, the elevated levels of pro-inflammatory C-reactive protein were found to negatively correlate with cognitive ability measured by a continuous performance test [87]. Even after the clearance of a SARS-CoV-2 infection, both systemic inflammation and neuroinflammation were detected, including increased pro-inflammatory cytokines in the CSF as well as myelin loss and microglial activation [88]. All these neuroimmune reactions have been associated with impaired cognition [89] and may additionally contribute to neurologic complications in long COVID patients [90].

Aberrant autoimmune responses (Fig. 4, B) triggered by SARS-CoV-2 have been proposed as another potential underlying mechanism for long COVID pathology [91–93]. This complication may induce targeted long-term tissue damage and therefore represents the major concern in clinical outcomes [94]. Produced in the course of an infection, the autoimmune antibodies have been shown to cross-react with proteins of the heart, brain, and blood vessels, causing chronic pathological phenotypes in COVID-19 patients [92, 95, 96]. Multiple studies have found elevated levels of autoantibodies in COVID-patients compared to uninfected controls [91, 97, 98]. A recent longitudinal study has demonstrated that 40% of patients had positive antinuclear antibodies 12 months following a SARS-CoV-2 infection [99, 100]. Results from two studies that specifically assessed autoantibodies in the long COVID patients have shown a persistence of postinfectious autoimmune processes [90, 101]. Autoantibodies linked to vasculature and thrombotic factors as well as antineuronal antibodies may both directly impact the CNS and lead to the development of long COVID. Autoimmunity can trigger an impairment of neurological functions, drive inflammatory processes and neuroinflammation, and induce neurodegeneration.

Another hypothesis suggests that a persistent SARS-CoV-2 infection (Fig. 4, C) may be responsible for long COVID symptoms, which, in addition to direct viral damage, may support chronic inflammation, and lead to immune-mediated tissue damage. The persistence of the virus may induce an ongoing immune stimulation and trigger chronic inflammation that, in turn, may cause cognitive impairments including diminished memory and executive functions. The viral RNA was detectable between 14 and 17 days [102, 103], suggesting a persistent infection in these patients. The extended duration of viral RNA detectable in the lung, respiratory tract, and feces has been reported, despite the absence of the virus in other clinical samples such as the sputum or nasal secretions [102, 104–106]. Though it has been shown that SARS-CoV-2 could infect the CNS, it is not currently clear if SARS-CoV-2 is capable of establishing a viral reservoir within the CNS. Only few reports demonstrated the detection of viral RNA in the CSF of long COVID patients [107], and a systematic evaluation of the CSF samples from a large cohort of patients with long COVID symptoms is required to shed light on this issue.

It has been suggested that some of the detrimental effects may be additionally caused by the direct action of the free viral spike protein, which can act either alone or in concerted action with other inflammatory mediators [108]. The spike protein could directly damage different types of cells, what could be particularly crucial for the CNS cells. The pathogenic effects may include direct injury and stimulation of the peripheral nerves [109] as well as the stimulation of production and release of inflammatory and vasoactive factors [110], such as the platelet-activating factor [111]. Especially crucial effects could be induced when the spike protein enters the brain or would be expressed in neuronal and glial cells, activating microglia and leading to neuroinflammation and neurodegeneration [108]. Such neurocognitive damage could be harmful in vulnerable elderly persons and those with minimal cognitive impairments.

The Cytomegalovirus (CMV) may represent another viral candidate (Fig. 4, D), which may contribute to the severity of COVID-19 and play a potential role in the development of long COVID [9]. The Cytomegalovirus is a ubiquitous and persistent herpesvirus with lifelong latency and immunomodulatory features, whereby the proportion of CMV-seropositive people increases with age [9, 112, 113]. The association of CMV serostatus with a clinical outcome of COVID-19 implies a role for a CMV-induced immune system remodeling in the pathogenesis of a SARS-CoV-2 infection [114]. CMV-reactivation may be responsible for chronic inflammation that often persists in patients with long COVID, who still have enduring symptoms even after SARS-CoV-2 is no longer detectable [115, 116].

The inflammatory status may itself lead to a reactivation of latent CMV infection, boosting neuroinflammation and contributing to the impairment of cognitive function [9, 113, 117]. The association between a CMV infection and cognitive decline in the general population has been confirmed in multiple studies [118–122]. Being a neurotropic virus, CMV induces CNS inflammation and may enter the brain either through disrupted BBB or by transfer via peripheral nerves [123]. The substantial sites of CMV localization were found in the brainstem, diencephalon, and basal ganglia [124], suggesting that CMV may itself or in combination with SARS-CoV-2 be responsible for CNS damage. Other studies found a depression-specific association of CMV with a reduced resting-state connectivity, white matter integrity, and gray matter volumes [125–127], indicating that CMV may induce structural and functional brain changes to a greater extent in elderly people with depression [16]. Thus, it is conceivable that a CMV infection may contribute to long COVID pathology by supporting immunosenescence, neuroinflammation, and by triggering persistent neurological symptoms, particularly in the older population.

Aberrant mitochondrial function has been hypothesized to be another important mechanism contributing to the development of the long COVID symptomology (Fig. 4, E). The experimental observations demonstrated that SARS-CoV-2 can raid mitochondria and use their functional capacities for its own survival [128]. Due to the replicative activity of the virus in the mitochondria, their metabolic ability may be changed, and elevated inflammatory reactivity occurs. The experimental evidence confirmed the mitochondrial dysfunction including the inability to generate sufficient adenosine triphosphate (ATP) and worsening symptoms in a COVID-19 infection [129, 130]. Such symptom as fatigue, but also other long COVID abnormalities, could be explained by the reduced tissue oxygen supply, owing to hypercoagulation and vascular dysfunction as well as to mitochondrial disturbances, thus disrupting critical cellular bioenergetics.

Mitochondrial failure together with hypercoagulation and chronic inflammation may lead to microthrombosis with an increased risk of stroke—thus contributing further to the pathology of long COVID. Microvascular damage, stroke, bleeding, and hypoxia-induced impairments contribute to brain tissue destruction, neuroinflammation, and neuronal loss, leading to neurodegeneration [84]. Endothelial dysfunction and coagulopathies (Fig. 4, F) are considered to be key mechanisms driving pathology during both acute disease and long COVID [131–134], with the SARS-CoV-2 spike protein potentially activated by clotting factors [135] and inflammation.

Dysbiosis has also been proposed as a contributory factor (Fig. 4, G) by allowing, through a disruption of the gut barrier integrity, a more easier transmission and dissemination of SARS-CoV-2 and the induction of systemic inflammation [136]. Changes in the gut microbiome, including depleted symbionts and gut dysbiosis after a SARS-CoV-2 infection, have been found to persist even after virus clearance and a resolution of clinical symptoms [137]. Due to the existing link between the microbiome, inflammation, and neuropsychiatric disorders [138–140], dysbiosis may also play a role in the genesis of long COVID. Further investigations are needed in order to better understand the neuroimmune effects of microbiome and its contribution to neuropsychopathology. It seems indispensable to closely consider the important role of microbiome in the regulation of neuroimmune interactions with the brain via “gut-brain axis” to better understand the pathophysiological mechanisms of long COVID.

Thus, we can conclude that different potential mechanism–some of which we summarized in this section (Fig. 4, A-G)—may operate in various combinations following a SARS-CoV-2 infection and thus contribute to long COVID. This is correspondingly reflected in the heterogeneous manifestations of long COVID symptoms. Nevertheless, the inflammation seems to be a core component in all these putative mechanisms, and both central and systemic inflammation may induce disturbed neuroimmune responses and lead to persistent physiological and neurological alterations, especially but not exclusively in an older population.

## Search for therapeutic strategies: modulatory effect of bioactive nutritional compounds and multimodal benefits of exercise on neuroimmune responses

There is no doubt yet, that following a COVID-19 recovery, numerous patients will require—due to the wide-ranging long COVID impairments—a targeted therapy and a long-term care. It is therefore vitally important to be aware, which of the most probable pathogenic mechanisms could be essential as a target for the beneficial therapy. On the other hand, it is also of a great importance to find generalized interventional solutions—such approaches that may allow for the treatment of long COVID in a holistic manner in order to mitigate its negative effects and improve quality of life.

As SARS-CoV-2 induces hyperstimulation of the neuroimmune system in the acute phase of infection and the persistent inflammation has been recognized as a core mechanism underlying the chronical phase, an immunomodulatory therapy targeting inflammatory processes may be the most beneficial to address the long COVID syndrome. In this section however, we will not discuss such therapeutic methods as an immunosuppressive therapy and plasmapheresis or other solely medical solutions, but rather shortly consider immunomodulatory interventions, including nutrition and physical exercise. These treatment modalities for the long COVID syndrome represent a low-risk options and are especially important for elderly people, who have also a rather increased medicamental load. In addition to the noninvasiveness of these interventions, they should be readily accessible and could be easily integrated in the every-day life.

These, at a first glance a very simple recommendations, could involve such long-term interventions for rehabilitation as controlled physical exercises and adequate sleep, musical therapy and meditation, and the ingestion of probiotics and other nutritious foods with anti-inflammatory characteristics. Such therapeutic options can decrease the propagation of biological, physiological, and psychosocial stressors, which are responsible for neuroimmune activation and inhibit in this way the triggering of unbalanced inflammatory responses from immune substrates such as microglia, which have been initially primed by the COVID-19 infection [8, 141].

Since microbiome may have a modulatory impact on inflammation, the ingestion of probiotic supplements was examined in 6 randomized studies and was demonstrated to be a safe therapeutic treatment for both young and elderly participants [142]. Through the modulation of the immune system and inflammatory responses, probiotics have been shown to be effective in the attenuation of gastrointestinal and upper respiratory infections, also in older people and highly stressed adults [143]. The immunomodulating ability of probiotics was mostly linked to their regulatory function on inflammatory and anti-inflammatory cytokines [144, 145], providing potential to mitigate the negative effects of SARS-CoV-2 on an overactivation of the immune system or to elevate intensifying preexistent vulnerabilities. Additionally, the term “psychobiotic” has been proposed that “refers to a live organism that, when given in sufficient amounts, improves symptoms of psychiatric illness” [8]. Multiple evidence implies that psychobiotics can play an important role in improving states of anxiety, depression, and chronic fatigue symptoms [139, 146]. Various mechanisms have been proposed on how probiotics may impact the CNS, involving the regulation of the microbiome-gut-brain axis, alterations in the signaling through the vagus nerve, the spinal cord, and the positive modulation of endocrine and immune systems [8, 146]. As some probiotics have demonstrated anti-inflammatory effects, they were proposed for therapeutic application to diminish the neuroinflammation [145]. Further studies are required to prove the effectiveness of probiotics in mitigating long COVID symptoms.

The ability to modulate the immune response and alleviate the neuroinflammation caused by SARS-CoV-2 have been shown through the therapeutic usage of high doses of melatonin [147]. Furthermore, vitamin D has been proposed to down-regulate the negative effects of neuroinflammatory mediators and to have other immunomodulatory and anti-inflammatory effects, mitigating detrimental effects of COVID-19 and its possible neurological consequences in the post-infectious phase [148]. However, clinical trials elucidating the efficacy of melatonin and vitamin D in the prevention and management of the long COVID are still missing and should be actively encouraged.

Some bioactive compounds and natural antioxidants were proposed to have the beneficial modulatory effects on COVID-19 [108, 149, 150]. Flavonoids, isolated from green plants and seeds possess effective anti-inflammatory, anti-oxidant, and powerful cytoprotective characteristics [151]. Such flavonoids as quercetin and luteolin have been identified as the beneficial players against SARS-CoV-2 exhibiting broad antiviral effects. Moreover, luteolin can reduce the activation of microglial and mast cells [152–154] and inhibit signaling pathways involved in the activation of inflammasome [155]. These neuroprotective characteristics allow luteolin to counteract the neuroinflammation [156–158], preventing cognitive dysfunctions [159–161] and brain fog [162, 163], what makes these bioactive natural components to promising therapeutic agents in respect to long COVID.

Meditative practices represent another kind of useful, moderate, and low-cost intervention that may contribute to the mitigation of long COVID symptoms. In a variety of studies, the outcome of meditation has been associated with anti-inflammatory cytokine activity [164–168], as has been reported in numerous systematic reviews [169, 170]. Meditation has been shown to cause neural reorganization, “re-modulation, and re-regulation” of the neuroimmune responses [171, 172]. These studies have also demonstrated that mindfulness meditation has been associated with a number of changes toward the inhibition of inflammatory processes and may be applied in the treatment of long COVID [173].

The mind-body intervention, including meditation demonstrated a decrease in the levels of C-reactive protein and the downregulation of cytokine receptors [174]. Another study found a significantly reduced expression of pro-inflammatory cytokines, with a shift toward the secretion of anti-inflammatory cytokines [167]. Intervention combining meditation and yoga has normalized levels of the pro-inflammatory TNF-α [175]. Increased concentrations of anti-inflammatory IL-10 and a decrease in levels of pro-inflammatory IL-12 were found as result of meditation [164–167]. In a meta-analysis study [176], it has been revealed that mindfulness-based interventions generated significant positive effects on cytokine blood levels related to low-grade inflammation. Another meta-analysis study found that meditation resulted in the decrease of C-reactive protein level and the stabilization of blood pressure [170], concluding that meditation may leads to the modulation of essential physiological markers.

The mind-body technics have also been demonstrated to induce a neural reorganization in practitioners [171], therefore these interventional methods are applicable to patients as a therapy for symptoms related to the brain and cognitive dysfunctions as well as support for psychosocial complications [177, 178]. Some studies investigating effects of meditation on neurotransmitters and immune profiles of meditators [169] found various wide-ranging neuroimmune benefits by applying meditation on a regular basis. Meditation may help patients by the modulation of pro-inflammatory to anti-inflammatory responses and/or by decreasing the over-activation of a sympathetic nervous system over the relaxation process [166]. Such stabilizing parasympathetic responses may be trained and translated easily to daily life to combat the long COVID disease [172].

Music represents another (mostly overlooked) non-invasive approach that may contribute to the mitigation of post-COVID symptoms. The results of a meta-analysis study suggest that music can have modulatory effects on the cytokine levels, in particular by inducing the reduction of the IL-6 concentration in the blood. Additionally, the modulation of stress-induced neuroimmune responses has been demonstrated, also to the pathogenic stress initiated by the viral infection [56, 179]. Through the regulatory modulation of the function of the HPA-axis and concentration of IL-6, music positively influenced the immune system during acute stress [180, 181].

Music has demonstrated its positive effects in the cerebrovascular disease through stimulation of the parasympathetic nervous system, decreasing levels of adrenaline and noradrenaline as well as pro-inflammatory TNF and IL-6 cytokines [182, 183]. Multiple studies have proven a positive effect of music on the immune function, and decreased cortisol levels correlated with this effect. Elevations in salivary IgA-concentrations and IL-1 levels have been related to decreased cortisol concentrations [181, 184, 185]. There are also reports that music can activate the production of neurotransmitters and hormones, contributing to anti-tumor signaling and T-cell proliferation [186, 187]. Thus, such studies support the concept that music may beneficially modulate neuroimmune interactions and positively influence physiological and mental processes [179]. Therefore, applying music therapy for long COVID patients may be a useful supportive intervention, particularly in the elderly, frail people.

Regular physical activity may trigger positive alterations in the peripheral and brain immune systems, inducing anti-inflammatory conditions and supporting a homeostatic milieu as well as limiting the adverse effects of COVID-19 infection. The beneficial effects of exercise have been demonstrated in the early studies on inflammatory markers, by changes in the expression and affinity of some essential monoamine receptors, and an improved synaptogenesis [188, 189]. The reciprocal cross-interactions between neurotrophic and growth factors, where levels of one factor influence the production of the others, could be responsible for the exercise-induced rebalancing of these factors and the inhibition of inflammation [190] inducing positive effects on neurogenesis, neurotransmission and vascularization [191, 192]. Consequently, moderate exercise interventions may improve mental health, the psychological balance and mood—partly due to the improvements in the expression and release of the neurotrophic and growth factors [193, 194].

The positive effects of regular exercise on the brain were found to be related to restored neurotransmission and remyelination, to the refining of BBB-integrity, and to the improvement of the immune responses [5, 195] as well as to mitigating effects on chronic inflammation and autoimmunity [196, 197]. The improved BBB-permeability was reached through changes in proteins of the tight junctions, in the supporting activity of surrounding astrocytes, and in oxidative capacity of microglia, as well as by decreasing of inflammation [198, 199]. Exercises were also shown to reduce inflammation and to suppress microgliosis by the elevation of the anti-inflammatory cytokines’ levels of [200].

It has been demonstrated that physical activity may buffer the effects of psychosocial stress induced by COVID-19 and improve mental and physical health among the population [5, 201]. Therefore, regular physical exercise may possibly mitigate mental and psychological impairments related to the long COVID syndrome by improving the neuroimmune homeostatic conditions, including balanced interactions between neuromodulatory cytokines and neurotransmitters, opioids, and neurotrophic and growth factors. Despite certain evidence of the beneficial effect of exercise, there is still a need for optimal programs that could help individuals with persistent long COVID symptoms. Controlled physical interventions can have positive immunomodulatory effects on the disturbed neuroimmune responses and can be applied as a post-infectious intervention to improve brain health. The published recommendations accentuate the necessity of “symptom-titrated physical activity and tailored exercise in rehabilitation” emphasizing that proper and tailored exercise may be a promising therapeutic intervention for mitigating the long COVID symptoms [202]. This will help affected people—particular of old age—to achieve a more effective and faster recovery, increase their autonomy, functional ability, and general quality of life.

## Conclusions

It is not yet clear what impact the current pandemic will have on human physical and mental health in the long term. The far-reaching global effects may follow in the next few years, but may also be first evident decades later. The long-term neurological and psychiatric consequences may be related to the virus itself or to the associated secondary stressors, such as isolation, loneliness, feelings of lack of control, anxiety and a loss of social connectivity. This combination of pathogenic and psychosocial effects may jointly influence the balanced functioning of the neuroimmune system, especially in vulnerably aged people.

In fact, the pandemic is expected to have a particularly drastic impact on the elderly persons, who already have repeatedly experienced the pathogenic and psychosocial impacts over the course of their lives and are primed by their own stress history. On the other hand, it could be speculated that as the years went by, the currently young population who have had a mild or asymptomatic course of COVID-19 would later have a distinctive trajectory of their aging process, because they also had experienced a type of priming by the infection with the SARS-CoV2 previously in the lifespan. Age-related neuroinflammation and immunosenescence may then serve later as triggering factors to the earlier onset and more detrimental outcome of neuropsychiatric and neurogenerative pathological conditions. Additionally, physical, pathogenic, and environmental stressors experienced during the lifespan may act on the neuroimmune cellular substrates primed by SARS-CoV-2 in the past, contributing together with immunosenescence and neuroinflammation to more intense and unremitting pathological conditions in the future.

In fact, the whole spectrum of potential long-term neurological consequences of COVID-19 have not yet been realized. But we can already witness a significant increase in such medical conditions as depression, anxiety, insomnia, and eating disorders, which could be mirrored by a similar increase in dementia, and motor and cognitive neurodegenerative disorders in the coming years. Despite intense research currently, unfortunately, we are still not able to predict, who will develop the long-term complications, how long they will persist, if they will entirely resolve, and–most importantly–if they are preventable.

Therefore, it is important to understand the multidimensional interactions between psychosocial stressors, immunosenescence, inflammaging, neuroinflammation, and SARS-CoV2, which may shed important light on the molecular and neuroimmune mechanisms of response to COVID-19, and open novel ways for therapeutic and behavioral interventions. Important future research perspectives towards a better understanding of mechanisms for possible neuroimmune modulation, including behavioral interventions, exercise, and psychotherapy, will be fundamental to decrease disability due to the long COVID in order to return individuals to full physical and mental health as well as to the “normality” we used to know before this pandemic.

## Data Availability

Not applicable.

## List of abbreviations

COVID-19: coronavirus disease 2019
SARS-CoV-2: severe acute respiratory syndrome coronavirus 2
CMV: Cytomegalovirus
CNS: central nervous system
IL: interleukin
TLR: toll-like receptor
TNF: tumor necrosis factor
RNA: ribonucleic acid
IFN: interferon
TLR: pattern recognition receptors
SASP: senescence associated secretory phenotype
ROS: reactive oxygen species
CD: cluster of differentiation
MHC: major histocompatibility antigen
Ig: immunoglobulin
DNA: deoxyribonucleic acid
HPA: hypothalamic-pituitary-adrenal
CRH: corticotropin-releasing hormone
ACTH: adrenocorticotropic hormone
BBB: blood-brain barrier
ACE2: angiotensin-converting enzyme 2
NOX2: nicotinamide dinucleotide phosphate oxidase 2
CSF: cerebrospinal fluid
RT-qPCR: quantitative polymerase chain reaction with reverse transcription
ATP: adenosine triphosphate
